# Survival benefit of surgical resection after first-line triplet chemotherapy and bevacizumab in patients with initially unresectable metastatic colorectal cancer

**DOI:** 10.1186/s12957-020-01930-8

**Published:** 2020-07-08

**Authors:** Mahmoud A. Elshenawy, Ahmed Badran, Ali Aljubran, Ahmed Alzahrani, M. Shahzad Rauf, Abdelmoneim Eldali, Shouki Bazarbashi

**Affiliations:** 1 Medical Oncology Section, Oncology Centre, King Faisal Specialist Hospital and Research Centre, PO Box 3354, Riyadh, 11211 Saudi Arabia; 2 Clinical Oncology Department, Faculty of Medicine, Menoufia University, Shebin El Kom, 32511 Egypt; 3 Clinical Oncology Department, Faculty of Medicine, Ain Shams University, Cairo, 11591 Egypt; 4 Department of Biostatistics, Epidemiology and Scientific computing, King Faisal Specialist Hospital and Research Centre, PO Box 3354, Riyadh, 11211 Saudi Arabia

**Keywords:** Colorectal cancer, Triplet chemotherapy, FOLFOXIRI-Bevacizumab, Surgery

## Abstract

**Abstract:**

**Background:**

Surgical resection of metastatic disease in patients with initially non-resectable colorectal cancer (CRC) has improved overall survival. Intensified chemotherapy regimens have increased the probability of converting unresectable metastasis to resectable. Here, we report the result of combining intensive chemotherapy (triplet) and surgical resection of metastatic lesions in patients with metastatic CRC.

**Patients and methods:**

Patients with unresectable metastatic CRC were enrolled in phase I/II trial of triplet chemotherapy consisting of capecitabine, oxaliplatin, irinotecan, and bevacizumab. Patients were given 5–8 cycles induction chemotherapy of the above regimen followed by maintenance capecitabine and bevacizumab until disease progression, unacceptable toxicity, or patient request. All patients were assessed at a multidisciplinary conference for possible surgical resection of their metastatic disease at the time of inclusion in the trial and 2 monthly intervals thereafter.

Patients who underwent R0 resection of their metastatic disease received adjuvant oxaliplatin and capecitabine to complete a total of 6 months of chemotherapy.

**Results:**

Fifty-three patients were enrolled. The median age was 52 years (range 23–74), 29 (55%) were males, ECOG PS 0-1 was 13 (66%), 11 (42%) had a right-sided tumor, 29 (55%) had resection of their primary tumor, 22 (42%) had a single metastatic site, and 8 (15.1%) had a liver-limited disease.

Thirteen patients (24.5%) underwent surgical resection of residual metastatic disease +/− the primary tumor with 10 (18.9%) of them were R0.

The surgical group had a higher incidence of males compared to the non-surgical group (69.3% vs 47.2%, *p* = 0.2), equal performance status, lower median number of metastatic sites (1 vs 2, *p* = 0.09), higher mutant Kras (53.8% vs 34.2%, *p* = 0.3), and higher response rate (84.6% vs 56.2%, *p* = 0.3).

With a median follow-up duration of 89 months, the median PFS for the whole group was 16.1 months [95% confidence interval (CI) 9.1–20] and the median OS was 28.2 months (95% CI 22.5–53.3).

The median PFS for the surgery group was 18.9 months (95% CI 12.6–not reached) compared to 9.6 months (95% CI 7.0–18.3) for the non-surgical group, log-rank *p* = 0.0165. The median OS for both groups was not reached (95% CI 53.3–not reached) and 23.2 months (95% CI 17.0–28.4) respectively, log-rank *p* = 0.0006. Five-year PFS and OS for the surgery group were 46.2% and 67.6% respectively.

**Conclusions:**

Patients with unresectable metastatic CRC and fit for triplet chemotherapy should have the benefit of combining this intensified regimen and surgical resection of their metastatic disease if possible.

**Trial registration:**

Clinicaltrials.gov, NCT01311050, registered March 6, 2011, retrospectively registered.

## Introduction

Colorectal carcinoma (CRC) is the third most common malignancy worldwide and the second most common cause of cancer-related death in men and third in female [1]. A significant percentage of patients have metastatic disease at initial presentation, with lung and liver being the most common sites of metastasis. Most of these patients have unresectable metastatic lesions, rendering the disease incurable [2]. Synchronous liver metastases have been reported in approximately 25% of patients diagnosed initially as colorectal cancer, while approximately 50% develop liver metastases during their disease course [3].

Chemotherapy has been proven to improve survival in patients with unresectable metastatic CRC.

The 5-year survival rate in patients with unresectable metastatic disease is close to 10–20% [4–7].

Triplet chemotherapy regimens combining fluoropyrimidines, oxaliplatin, and irinotecan with or without biologics have shown improved efficacy compared to doublets with higher response rate, longer progression, and overall survival [8–10].

Surgical resection of metastatic disease in patients with initially non-resectable metastasis has shown improved overall survival compared to those who could not undergo resection in many retrospective series. Most of the published reports have evaluated the benefit of liver resection in such circumstances [11, 12]; however, resection of non-liver metastasis including peritonectomy with or without hyperthermic intraperitoneal chemotherapy (HIPEC) has also shown improved survival compared to no surgery in both single-arm and randomized trials [13–15].

The probability of converting unresectable metastasis to resectable has been shown to be more likely with triplet chemotherapy than with the standard doublet regimens [8, 16–19]. Most of these trials have concentrated on resection of liver metastasis.

We previously published the results of our phase I/II trial of a triplet consisting of capecitabine, oxaliplatin, and irinotecan with bevacizumab in patients with advanced CRC [20].

Here, we report the post hoc analysis of the efficacy of combining intensive chemotherapy (triplet) and surgical resection of metastatic lesions in the above cohort of patients.

## Methods

### Study design

The present report represents a post hoc analysis of the previously published phase I/II trial of the triplet therapy in patients with unresectable metastatic or locally advanced colorectal cancer with analysis of the results in patients who underwent surgical resection.

### Patients

Patients with metastatic CRC not amenable to surgical resection were enrolled in a phase I/II trial of triplet chemotherapy consisting of capecitabine, oxaliplatin, irinotecan, and bevacizumab [20]. Inclusion criteria included age more than 18 years, histologically confirmed CRC adenocarcinoma, no prior chemotherapy or targeted therapy for metastatic disease, Eastern Cooperative Oncology Group performance status (ECOG PS) 0-2, measurable disease as defined by response evaluation criteria in solid tumors (RECIST) V1.1 [21], and adequate organ function (absolute neutrophil count (ANC) ≥ 1.5 × 10^9^/l, platelet count ≥ 100 × 10^9^/l, normal serum bilirubin, serum transaminases ≤ 2.5 times the upper limits of normal (ULN), normal serum creatinine, and urine dipstick for proteinuria ≤ 2+). Patients who had prior adjuvant oxaliplatin or fluoropyrimidine chemotherapy were eligible if the last chemotherapy was ≥ 12 months. Patients were considered ineligible if they had any of the following criteria: central nervous system metastasis, prior malignancy within 5 years (except for adequately treated non-melanoma skin cancer or in situ cervical cancer), sever cardiovascular dysfunction, bleeding diathesis, major surgery within 28 days of starting therapy, active infection, uncontrolled hypertension, pregnancy or breastfeeding, and prior history of dihydropyrimidine deficiency.

### Treatment

The phase I part of the trial has been described earlier in the previous publication [20]. Based on the phase I part, the recommended doses for the phase II were capecitabine 1000 mg/m^2^ orally on days 1 to 14, oxaliplatin 130 mg/m^2^, irinotecan 150 mg/m^2^, and bevacizumab at 7.5 mg/kg of body weight, all on D1 of each cycle. Cycles were repeated every 21 days. Patients were given induction chemotherapy of the above regimen for 5–8 cycles followed by maintenance capecitabine and bevacizumab at the above doses till disease progression or unacceptable toxicity. All patients were assessed at a multidisciplinary conference for possible surgical resection of their metastatic disease at the time of inclusion in the trial and at 2 monthly intervals. Surgical resectability was at the discretion of the operating surgeon and according to standard surgical procedures. Patients with retroperitoneal lymph nodes were considered unresectable.

Patients who underwent R0 resection of their metastatic disease received adjuvant oxaliplatin and capecitabine to complete a total of 6 months of chemotherapy. Radiation therapy was not given in the pre- or post-operative setting.

### Statistics and efficacy endpoints

The statistical design of the phase I and II parts of this study was described earlier [20]. The number of patients planned for the phase II part of the trial was 46. All patients were assessed for response according to RECIST criteria V1.1 [21] by CT scans or MRI performed after the second, fifth, and eighth cycles of chemotherapy and every 2 months thereafter.

Progression-free survival (PFS) was calculated from the date of starting chemotherapy to the date of first documented disease progression, recurrence, or death from any cause. Overall survival (OS) was calculated from the start of chemotherapy to date of last follow-up or death from any cause. Tabulation and statistics were performed in the SAS statistical software application (version 9.4: SAS Institute, Cary, NC, USA). The Kaplan-Meier method was used to calculate PFS and OS. Calculation of *p* values used the log-rank test, and results were considered statistically significant if *p* is equal to or less than 0.05. The chi-square test was used to calculate the *p* value for the different factors between both groups.

## Results

### Patients’ characteristics

A total of 53 patients with metastatic or locally advanced unresectable CRC were enrolled on a phase I/II trial of combination chemotherapy with capecitabine, oxaliplatin, irinotecan, and bevacizumab (6 on the phase I part and 47 on the phase II part). Patients’ characteristics are illustrated in Table 1. Eight (15.1%) patients had liver-limited disease (LLD). Thirteen patients (24.5%) underwent surgical resection of residual metastatic disease +/− the primary tumor with 10 (18.9%) of them were R0. The other forty patients were deemed unresectable.

**Table 1:** Characteristics of 53 patients treated with the triplet chemotherapy regimen

Median age (range) (y)	52 (23–74)
Male/female [number (%)]	29 (55)/24 (45)
ECOG performance status [number (%)]	
0	7 (13)
1	35 (66)
2	11 (21)
Primary tumor site [number (%)]	
Colon	23 (40)
Rectosigmoid	21 (36)
Rectum	9(15)
Prior surgery for primary tumor [number (%)]	29 (55)
Prior adjuvant chemotherapy [number (%)]	6 (11)
Prior radiotherapy [number (%)]	0
Number of metastasis sites [number (%)]	
Single	22 (42)
Multiple sites	31 (58)
Metastasis sites [number (%)]	
Liver	35 (66.0)
Lung	22 (41.5)
Lymph nodes	21 (39.6)
Peritoneum	14 (26.4)
K-ras [number (%)]	
Wild-type	20 (37.0)
Mutated	20 (37.0)
Unknown	13 (26.0)

*Y* years

The characteristics of the 13 patients are illustrated in Table 2. Ten of them had synchronous metastasis, and 3 were metachronous. The surgical group had a higher incidence of males compared to non-surgical group (69.3% vs 47.2%, *p* = 0.2), equal performance status, lower median number of metastatic sites (1 vs 2, *p* = 0.09), higher mutated Kras (53.8% vs 34.2%, *p* = 0.3), and higher response rate (84.6% vs 56.2%, *p* = 0.3), Table 3. The type of surgical procedure performed in each of the 13 patients in addition to significant surgical complications is listed in Table 4.

**Table 2:** Characteristics of patients who underwent surgical resection (*n* = 13)

	No. (%)
Age (years)	
Median	50.5 (IQR 41–57)
Gender	
Male	9 (69.2)
Female	4 (30.8)
ECOG PS	
One	9 (69.2)
Two	4 (30.8)
Sidedness of the primary tumor	
Right	3 (23.1)
Left	10 (76.9)
Number of organs involved	
Median	1
Range	(1–2)
One	8 (61.5)
Greater than one	5 (38.5)
Site of metastasis	
Liver	5 (38.5)
Lung	1 (7.7)
LNs	6 (46.2)
Peritoneum	5 (38.5)
KRAS gene mutation	
Mutant	7 (53.8)
Wild-type	5 (38.5)
Unknown	1 (7.7)
Best response to XELOXIRI/A	
CR	1 (7.7)
PR	10 (76.9)
SD	2 (15.4)
Surgical resection margin	
R0	10 (76.9)
R1	3 (23.1)

*IQR* interquartile range; *XELOXIRI/A* xeloda, oxaliplatin, irinotecan, and avastin; *ECOGPS* Eastern Cooperative Oncology Group Performance Status; *CR* complete response; *PR* partial response; *SD* stable disease; *Primary* primary tumor resection

**Table 3:** Patients’ characteristics in surgical and non-surgical groups

Item	Surgical resection	No surgical resection	*p* value
Median age	50.5 (IQR 41–57)	54 (IQR 43–59)	0.8
Males %	9 (69.3%)	18 (47.2%)	0.2
Median performance status	1 (IQR 0.5–1)	1 (IQR 1–2)	0.3
% right-sided primary	3 (23.1%)	8 (21.1%)	0.8
Median number metastatic site	1 (IQR 1–2)	2 (IQR 1–3)	0.09
% mutant KRAS	7 (53.8%)	13 (34.2%)	0.3
% best response	11 (84.6%)	18 (56.2%)	0.3

*IQR* interquartile range

**Table 4:** Surgical procedures after the triplet chemotherapy regimen

No.	Surgical procedure	No. of chemotherapy cycles prior to surgery	Hospital stay (days)	Major surgical complications
1	Cytoreductive surgery [subtotal colectomy + bilateral oophorectomy + cholecystectomy] + HIPEC	8	30	
2	Cytoreductive surgery [abdominal wall resection + left hemicolectomy + appendicectomy] + HIPEC	13	13	Pancreatic leakage
3	Cytoreductive surgery [extended left hemicolectomy + distal pancreatectomy + small bowl resection] + HIPEC	12	29	
4	Segmental liver resection	10	7	
5	Cytoreductive surgery [subtotal colectomy + appendicectomy + cholecystectomy + splenectomy] + HIPEC	6	15	
6	Bilateral pulmonary metastatectomy	2	12	
7	High anterior resection	7	9	
8	Cytoreductive surgery [extended left hemicolectomy + splenectomy + appendicectomy + cholecystectomy] + HIPEC	9	17	
9	Anterior resection + liver metastatectomy	16	18	
10	Total proctocolectomy	7	13	
11	Anterior resection + liver metastatectomy	18	28	Hospital-acquired pneumonia
12	Sigmoidectomy + liver metastatectomy	20	16	
13	Liver metastatectomy	9	14	

*HIPEC* hyperthermic intraperitoneal chemotherapy

The median number of triplet chemotherapy cycles given prior to surgery was 5 (range 2–8). The median number of chemotherapy cycles given prior to surgery (induction triplet therapy + maintenance capecitabine and bevacizumab) was 9 (range 2–20). The median duration from the start of chemotherapy to surgery was 311 days (range 70–552).

### Efficacy analysis

Forty-five patients of the total 53 patients were evaluable for response, 2 (4.4%) patients achieved complete response (CR), and 27 (60%) achieved partial response (PR) for overall response rate (ORR) of 64%. Sixteen (31%) patients achieved stable disease (SD). The disease control rate was 95%. Eight patients were not evaluable for response for the following reasons: 4 withdrew consent, 2 were discontinued because of grade 4 toxicity, and 2 died before evaluation. Seven (15.5%) of the evaluable patients had a tumor shrinkage of ≥ 40% [22]. Two of the thirteen patients who underwent surgical resection achieved complete pathological response (pCR).

**Fig. 1:**
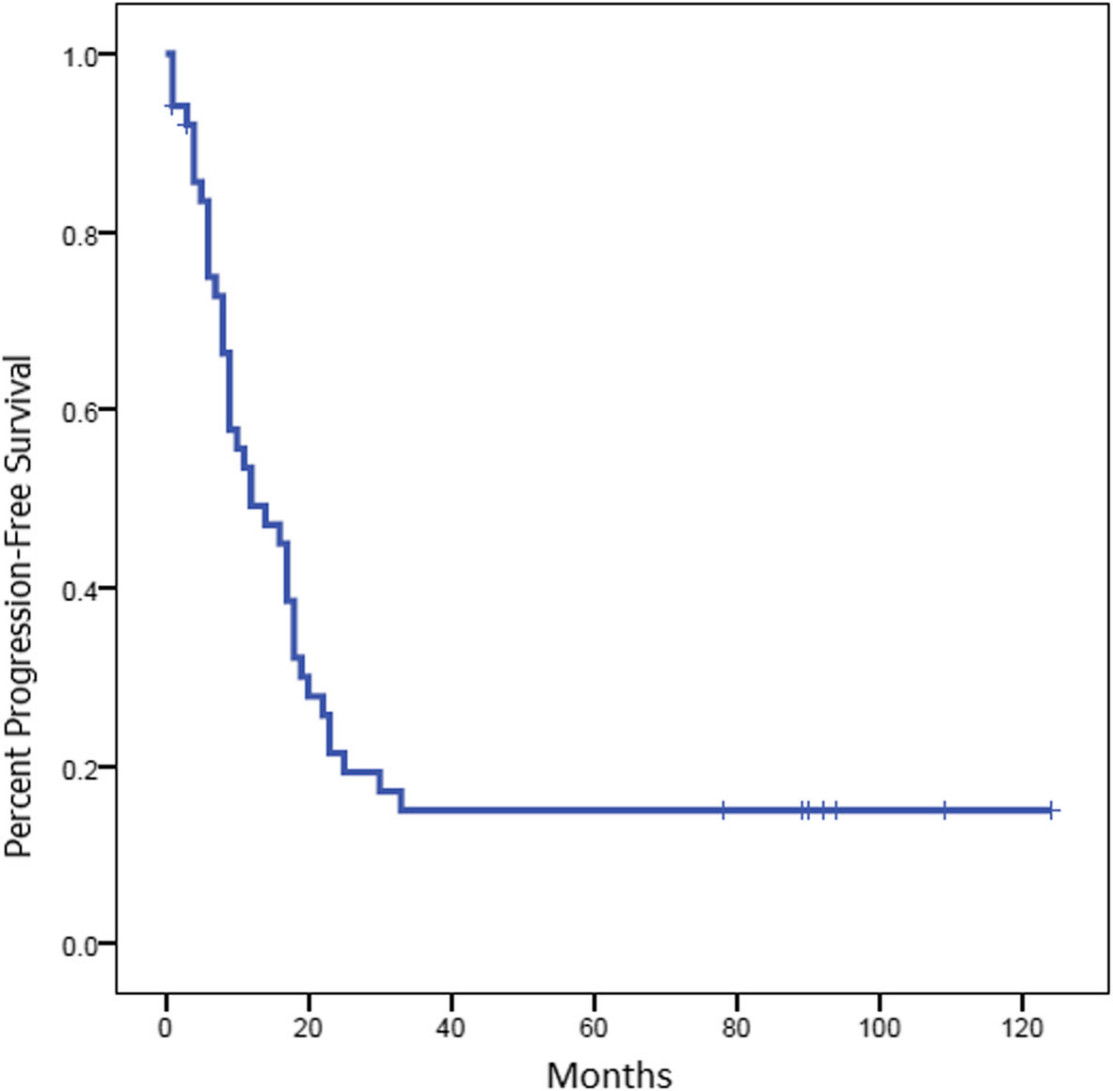
Progression-free survival (PFS) of the whole group

**Fig. 2:**
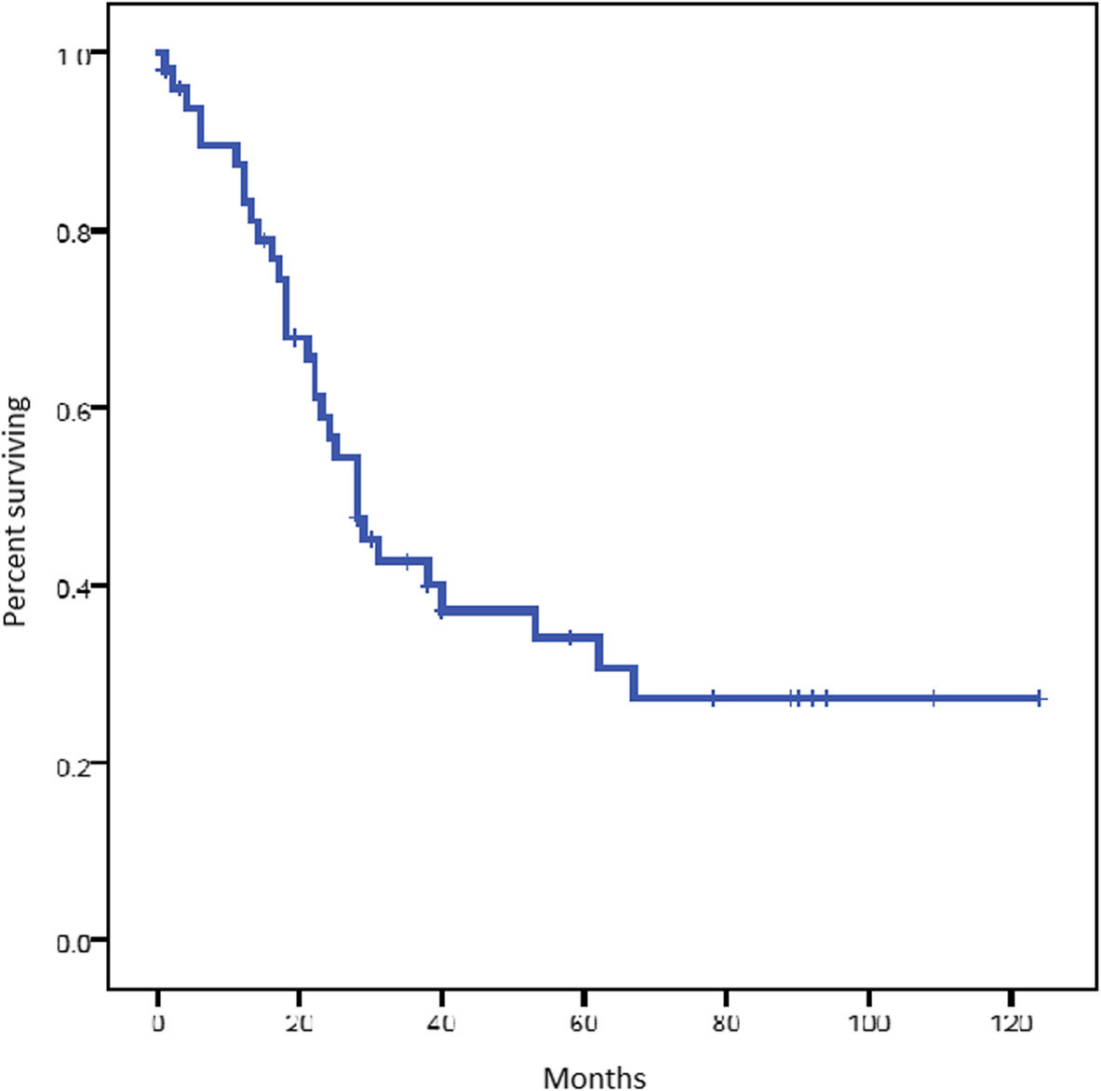
Overall survival (OS) of the whole group

With a median follow-up duration of 89 months, the median PFS for the whole group was 16.1 months [95% confidence interval (CI) 9.1–20] and OS was 28.2 months (95% CI 22.5–53.3) (Figs. 1 and 2).

**Fig. 3:**
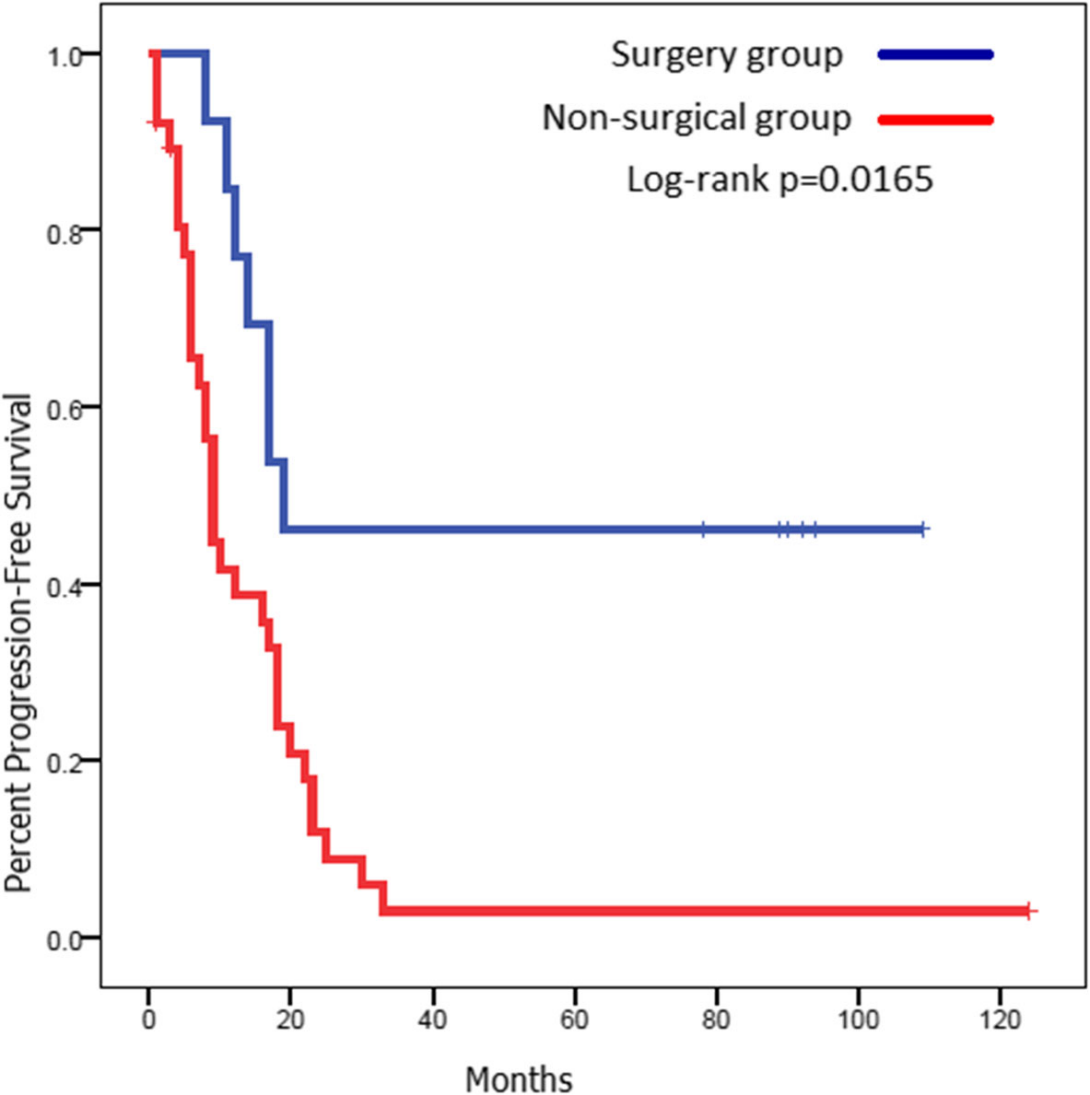
Progression-free survival (PFS) of the surgical vs the non-surgical groups

**Fig. 4:**
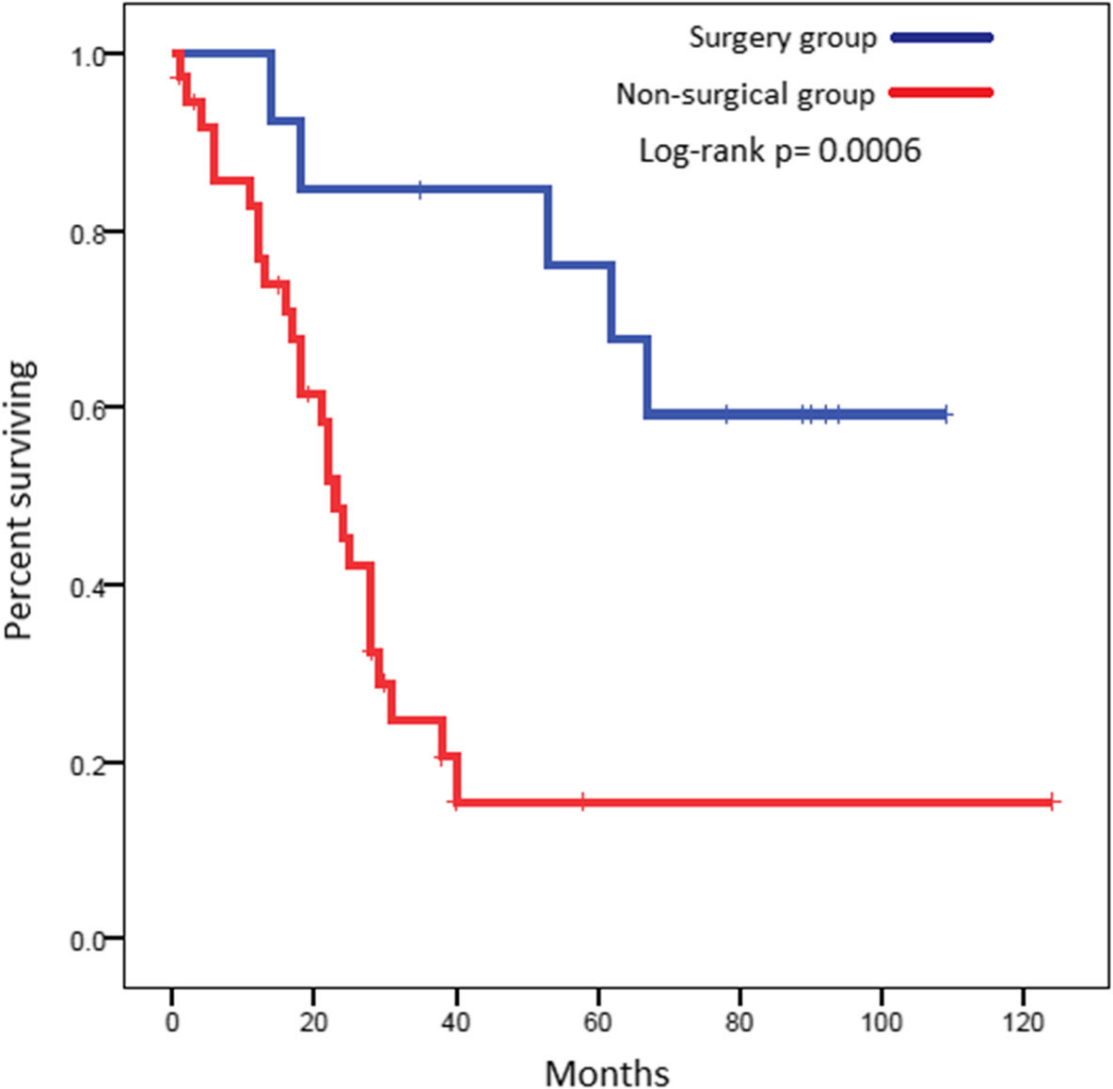
Overall survival (OS) of the surgical vs the non-surgical groups

The PFS for the surgery group was 18.9 months (95% CI 12.6–not reached) compared to 9.6 months (95% CI 7.0–18.3) for those who did not have surgery, log-rank *p* = 0.0165. The OS for both groups was not reached (95% CI 53.3–not reached) and 23.2 months (95% CI 17.0–28.4) respectively, log-rank *p* = 0.0006) (Figs. 3 and 4). Five-year PFS and OS for the surgery group were 46.2% and 67.6% compared to 15.4% and 3% in the non-surgical group respectively.

The toxicity and adverse events of the triplet regimen have been published earlier [20].

## Discussion

Surgical resection of metastatic disease of colorectal cancer has been shown in many trials to result in long-term disease-free and overall survival [11–15] and represents the only curative modality in this group of patients. The addition of targeted therapy to doublet chemotherapy regimens, in particular anti-EGFR therapy, has improved the resectability in patients with RAS wild-type phenotype with R0 resection ranging from 11.8 to 30.8% [17, 23, 24]. Triplet chemotherapy has also shown to increase the resectability rate in patients with unselected RAS phenotype. In a meta-analysis of 4 randomized trials with 1013 patients comparing triplet vs doublets regimens [25], the triplet chemotherapy did increase the R0 resectability with relative risk (RR) of 1.41 (95% CI 1.07–1.85). Liver R0 resection rate also increased with RR of 2.28 (95% CI 1.34–3.89). In this meta-analysis, the OLIVIA trial enrolled patients with only liver-limited disease (LLD) with higher response rate of 81% for the FOLFOXIRI-BEV vs 62% for FOLFOX-BEV. The overall resection rate was 61% vs 49%, and the R0 resection rates were 41% vs 23% respectively [10]. The R0 resection rate for the TRIBE trial was 15% [8]. The other 2 trials in the meta-analysis were the CHARTA which was published in an abstract form and the STEAM trial which reported liver resection rate of 17.2% [26, 27].

A pooled analysis published in 2017 of 11 trials with 889 patients utilizing FOLFOXIRI-Bevacizumab showed an overall resection rate of 39.1% (95% CI 26.9–52.8%) with an R0 resection rate of 28.1% (95% CI 18.1–40.8%) [28]. Out of those trials, three were all LLD while the mean incidence of LLD in the other 7 trials was 35.9% (range 18–52%). One trial did not report the percentage of LLD. The mean resectability rate in the 3 trials reporting non-LLD was 24.7% (range 15–32%).

The patient population in our trial constitutes a high-risk group with 21% performance status of 2, 11% with prior adjuvant chemotherapy, 37% KRAS mutant, and 58% multiple sites of metastasis. Five patients (9.4%) underwent liver resection (3 with resection of the primary), 5 (9.4%) had cytoreductive surgery plus HIPEC as part of their surgical management, and one had pulmonary metastatectomy. The remaining two had resection of initially unresectable primary tumor.

The median number of chemotherapy cycles in our patients was 9 (range 2–20). This goes with the prior finding of higher probability of secondary surgery with increasing number of cycles [29]. Despite relatively high number of chemotherapy cycles in the patients who underwent surgical resection, only 2 had major surgical complications with one developing pancreatic leakage and the other developing hospital-acquired pneumonia. This in contrast to the previous report by Karoui et al. where 54% of the patients who had equal or more than 6 cycles and underwent major liver surgery had surgical complications compared to 19% in those who had received less than 6 cycles (*p* = 0.047) [30]. Our lower rate of surgical complications is likely secondary to refinement of the surgical procedures and postoperative management.

It is likely that the addition of bevacizumab to intensive chemotherapy regimens does not increase the rate of resectability since this had not been proven elsewhere. On the other hand, the addition of antibody to epidermal growth factor receptor (EGFR) has shown an increase in the response and resectability rates at least in patients with unresctable LLD. In the UNICANCER PRODIGE 14-ACCORD 21 (METHEP-2) trial, the ORR for FOLFORINOX-Bevacizumab was 58% compared to 83% in patients who received FOLFORINOX-Cetuximab. The R0/R1 resection rates were 54% vs 63% respectively [31].

The relatively high resection rate with our triplet regimen resulted in an encouraging 5-year PFS of 46.2% and OS of 67.5%. This clearly indicates the benefit of combining intensive chemotherapy and surgery (for those who became resectable) as compared to chemotherapy alone where the 5-year PFS and OS were much lower at 3% and 15.4% respectively. This should be balanced against the higher toxicity rates of triplet regimens. The grades 3/4 toxicities of our regimen with oral capecitabine (reported earlier) [20] were unfortunately higher than infusional 5-fluorouracil regimens.

Our study had several limitations including a small number of patients, being a single-institution study, and post hoc nature of the analysis.

In conclusion, triplet chemotherapy regimens in patients with initially unresectable metastatic colorectal cancer yield a high rate of resection which results in long-term progression-free and overall survival in the resected group of patients. All eligible patients for such therapy should be given the benefit of this intensified chemotherapy regimens.

## Data Availability

The datasets used and analyzed during the current study are available from the corresponding author upon any reasonable request.

## Abbreviations

CRC: Colorectal cancer
ECOG: Eastern Cooperative Group Performance Status
PFS: Progression-free survival
OS: Overall survival
CI: Confidence interval
HIPEC: Hyperthermic intraperitoneal chemotherapy
RECIST: Response evaluation criteria in solid tumors
ANC: Absolute neutrophil count
IRB: Institutional review board
LLD: Liver-limited disease
CR: Complete response
PR: Partial response
ORR: Overall response rate
SD: Stable disease
pCR: Pathological complete response
RR: Relative risk
